# Epidemiology and management of 10,486 pediatric fractures in Shenzhen: experience and lessons to be learnt

**DOI:** 10.1186/s12887-022-03199-0

**Published:** 2022-03-29

**Authors:** Xin Qiu, Hansheng Deng, Qiru Su, Shuaidan Zeng, Shuai Han, Shicheng Li, Zhiwen Cui, Tianfeng Zhu, Gen Tang, Zhu Xiong, Shengping Tang

**Affiliations:** 1 Department of Pediatric Orthopedics, Shenzhen Children’s Hospital, Shenzhen, Guangdong China; 2 Zunyi Medical University, Zunyi, Guizhou Province People’s Republic of China; 3 China Medical University, Shenyang, Liaoning Province People’s Republic of China

**Keywords:** Pediatric, Fracture, Epidemiology, Pediatric trauma centers

## Abstract

**Purpose:**

To explore and analyze the causes and related influencing factors of pediatric fractures, and provide theoretical basis for reducing the incidence and adverse effects of pediatric fractures.

**Methods:**

This study retrospectively analyzed the epidemiological characteristics of fractures in pediatric aged ≤18 years old who were admitted to the our hospital between July 2015 and February 2020.

**Results:**

A total of 10,486 pediatric patients were included in the study, of whom 6961 (66.38%) were boys, and 3525 (33.62%) were girls. For the fracture incidence, age group of the 3-6 years reached the peak. 5584 (60.76%) children were operated upon within 12 h after admission. The top three types of fractures were the distal humerus (3843 sites, 27.49%), distal ulna (1740 sites, 12.44%), and distal radius (1587 sites, 11.35%). The top three causes of injury were falls (7106 cases, 82.10%), car accidents (650 cases, 65.72%), and clipping (465 cases, 5.37%). Fractures predominantly occurred between July and November (4664 cases, 48.87%) and on Saturdays and Sundays (3172 cases, 33.24%). The highest number of hospital visits occurred between 20:00 and 00:00 (4339 cases, 45.46%).

**Conclusion:**

For pediatric fractures, we should take appropriate and effective preventive measures to reduce the incidence of children’s fractures according to the distribution characteristics of age, gender, cause of injury, and fracture site.

**Supplementary Information:**

The online version contains supplementary material available at 10.1186/s12887-022-03199-0.

## Introduction

Fractures are very common in pediatric trauma and account for 10 to 25% of all fractures; moreover, the incidence of fractures has been increasing yearly [1–3]. Previous studies have reported the epidemiology of pediatric fractures. For example, from 1993 to 2006 in Malmö, Sweden, the incidence of fractures declined for girls, but not for boys [4]. In addition, data from Switzerland and Norway on long bone pediatric and adolescent fractures showed differences in fracture distribution with respect to sex and age [5, 6]. A retrospective study conducted by Rennie et al. in Edinburgh in 2000 showed a yearly incidence of 20.2 pediatric fractures per 1000 children [7]. The pediatric fracture sites also varied by country and region [8, 9]. A recent study conducted in India showed that elbow fractures are the most common pediatric fracture types [10], which was similar to the results of a study conducted in Hong Kong, China [11]. According to the 2015 Chinese National 1% Population Sample Survey, the population of children aged 0-17 years in China was 271 million, which accounted for 12.9% of the global pediatric population. With the development of urbanization, motorization, and the construction industry in China, the pattern of post-traumatic fractures in children and adolescents is likely to change. However, in China, no systematic studies have been conducted on the overall pattern and epidemiological trends of pediatric and adolescent fractures.

Additionally, the world’s first trauma center was founded in 1941 in Birmingham, England, whereas that in the United States of America (USA) was established in the 1960s by Cowley at the University of Maryland, and it was named the Shock Trauma Center. In 1971, Illinois became the first state in the USA to establish a regional trauma care system. In China, however, pediatric trauma medicine had a late start and is still exploring its way forward. Currently, the coverage of the emergency network in China is uneven, pediatric trauma centers (PTCs) are rare, there is a lack of complete regional trauma treatment coordination systems, the distribution of medical resources is unreasonable in most regions, and the link between pre-hospital and in-hospital emergency care is broken. Furthermore, with regards to the treatment of patients with severe trauma, the vast majority of hospitals still implement a multidisciplinary consultation model, and lack a substantively operating comprehensive pediatric trauma treatment team. This can lead to delayed in-hospital treatment and deprive patients with trauma of the best treatment opportunities. This study conducted an epidemiological analysis of pediatric trauma hospitalized in a Tertiary grade A hospital in children’s specialized hospital, which are beneficial to provide a reference for the subsequent construction of a pediatric trauma centers.

## Materials and methods

Between July 2015 and February 2020, we performed a retrospective analysis on 10,486 pediatric patients aged ≤18 years who were admitted to the orthopedic department of our hospital in Guangdong, China for fracture treatment. Patients were included with fresh fractures confirmed by plain radiography, computed tomography, and/or magnetic resonance imaging, and who were treated at our hospital within 14 d following the injury. After searching the medical record system of all patients with fractures, clinical and imaging data were analyzed according to their inpatient admission number. Data extracted included the cause of injury, sex, age, fracture site, interval between injury and hospital visit, concomitant trauma to other systems, year of admission, hospitalization cost, and hydrological and meteorological data at the time of injury.

The inpatients were grouped by age, as follows: age ≤ 730 d, infants; 730 d < age ≤ 2190 d, preschool children; 2190 d < age ≤ 4015 d, school children; 4015 d < age ≤ 6570 d, adolescents. The fracture sites were classified according to their anatomical location into skull, clavicle, scapula, proximal humerus, shaft of humerus, distal humerus, proximal ulna, shaft of ulna, distal ulna, proximal radius, shaft of radius, distal radius, carpal bones, metacarpal bones, phalanges of fingers, rib, sternum, vertebrae, pelvis, proximal femur, shaft of femur, distal femur, patella, proximal tibia, shaft of tibia, distal tibia, proximal fibula, shaft of fibula, distal fibula, tarsal bones, metatarsal bones, and phalanges of toes.

The causes of fractures were classified according to the mechanism of injury into seven first-level categories: daily-life injuries, road traffic injuries, sports injuries, abuse injuries, birth injuries, iatrogenic injuries, and unknown causes of fracture. The first-level categories were classified into second-level subcategories. Daily-life injuries were divided into falls, cuts, strains, sprains, twist injuries, clipping, furniture-related falls, crush injuries, falls from height, bunk bed falls, bites, and others. Road traffic injuries were divided into car accident injuries, bicycle falls, bicycle-spoke injuries, and falls from vehicles. Sports injuries were divided into slide falls, trampoline falls, single/parallel bar falls, skateboard falls, fitness equipment falls, basketball falls, rocking horse falls, falls while running, balance bike falls, ice skating falls, swing falls, ski falls, taekwondo falls, kick injuries, dance falls, playground falls, soccer falls, falls during physical education activities, jump rope falls, swimming-related injuries, martial arts falls, and others.

The interval between the patient’s injury and hospital admission was divided into < 4 h, 4-6 h, 7-11 h, 12-23 h, 24-47 h, 48-71 h, 72 h-5 d, and 6-14 d. Concomitant systemic trauma was divided into nervous, endocrine, circulatory, respiratory, digestive, urinary, and reproductive system trauma.

Based on the geographic area where the fracture occurred, we divided the administrative areas of Shenzhen into four regions: eastern Shenzhen (Longgang, Yantian, Pingshan, and Dapeng New Districts), western Shenzhen (Baoan, Guangming, and Nanshan Districts), northern Shenzhen (Longhua District); and southern Shenzhen (Futian and Luohu Districts). Other regions involved were Huizhou City, Guangdong Province; Dongguan City, Guangdong Province; and Hong Kong Special Administrative Region, China. This study was approved by the Ethics Committee of our hospital.

## Results

### Age and sex

A total of 10,486 pediatric patients were included in the study, with 6961 (66.38%) boys and 3525 (33.62%) girls. Boys had more fractures than girls in all age groups. The age groups with the highest and lowest number of fractures in boys were the preschool children and infant groups, respectively; whereas those in girls were the preschool children and adolescents groups, respectively.

Among the four patient age groups, the infants group had the lowest number of fracture cases (1227 cases, 11.70%), including 694 (56.56%) boys and 533 (43.44%) girls. Due to the rapid growth and development of children, the number of fracture cases gradually increased and peaked in the preschool children group (4380 cases, 41.77%), including 2719 (62.08%) boys and 1661 (37.92%) girls. Thereafter, the number of fracture cases showed a decreasing trend, with 3622 cases (34.54%) in the school children group, including 2478 (68.42%) boys and 1144 (31.58%) girls. The number of fracture cases in the adolescent group was 1257 (11.99%), including 1070 (85.12%) boys and 187 (14.88%) girls. Moreover, the sex ratio (males to females) for fractures increased with age, reaching a peak of 5.7:1 in the adolescents group (Table 1).

**Table 1:** Demographics of patients with 10,486 fractures

Parameter	Patients N(%)
Numbers	10,486
Age class	
Infants	1227 (11.70%)
Preschool children	4380 (41.77%)
School children	3622 (34.54%)
Adolescents	1257 (11.99%)
Sex	
Girl	3525 (33.62%)
Boy	6961 (66.38%)
Hospitalization expenses (RMB)	8142.68 ± 11,140.46
Hospital stays (day)	5.22 ± 7.63

### Fracture site

**Fig. 1:**
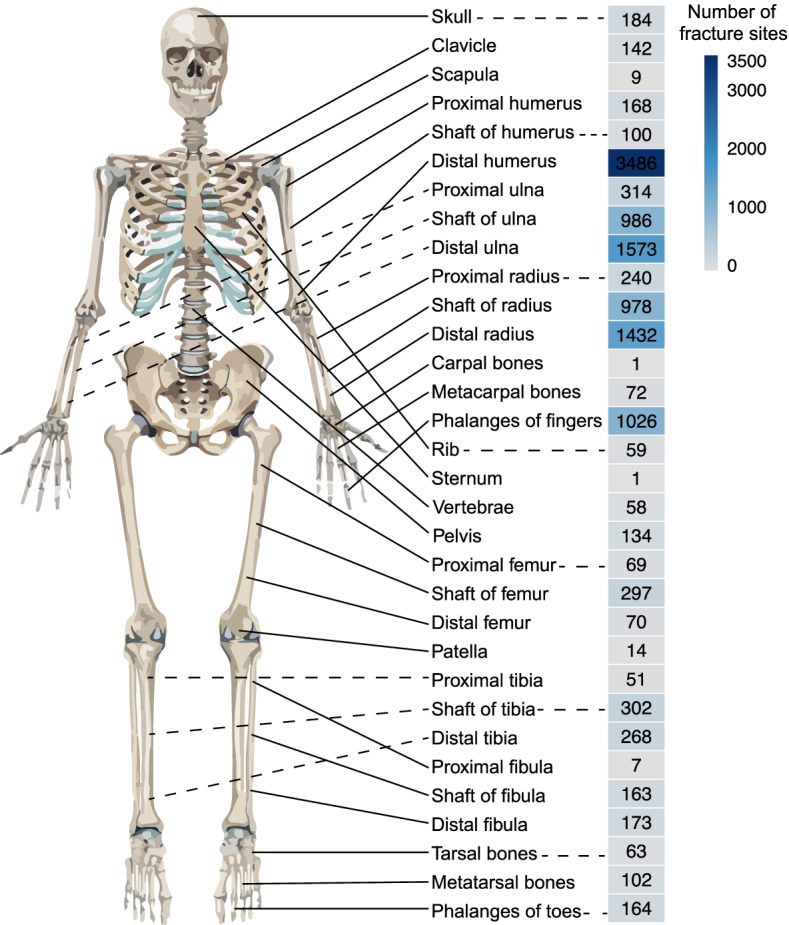
Sites and fracture patterns. This picture shows the common fracture sites in children of all age groups

There were 13,982 fracture sites among the 10.486 pediatric patients. The numbers of patients with 2, 3, 4, 5, 6, 7, and 9 concomitant fracture sites were 1014 (9.67%), 126 (1.20%), 30 (0.29%), 16 (0.15%), 6 (0.06%), 2 (0.02%), and 1 (0.01%), respectively (Fig. 1).

**Fig. 2:**
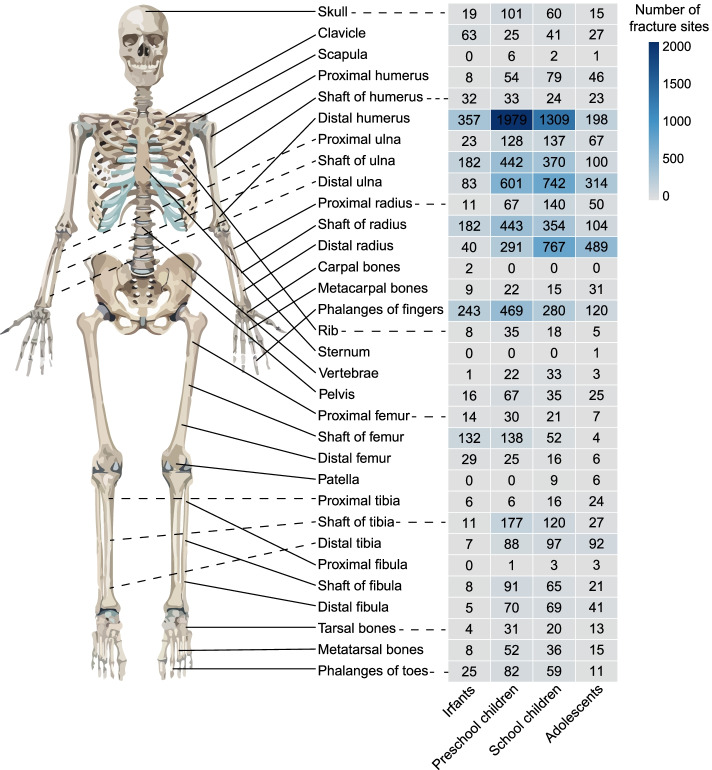
The epidemiology of traumatic fractures in all age range groups. This picture shows the characteristics of common fracture sites in the infants group, preschool children group, school children group and adolescent group

The most common fracture site was the distal humerus with 3843 sites (27.49%), which was similar in both boys and girls. Distal humerus fractures occurred most frequently in the preschool children group (1979 sites), and the number of such fractures tended to decrease with increasing age. Furthermore, distal ulnar fractures occurred in 1740 sites (12.44%), with a predominance in the school children group (742 sites). Distal radius fractures occurred in 1587 sites (11.35%), predominantly in the school children group (767 sites). Fractures of the phalanges of fingers occurred in 1112 sites (7.95%), mainly in the preschool children group (469 sites) (Fig. 2).

The retrospective analysis of the four calendar years from January 2016 to December 2019 indicated that distal humerus fractures (i.e., the most common fracture) peaked at 937 sites (24.38%) in 2018, which decreased to a trough of 758 sites (19.72%) in 2019. Moreover, distal ulnar and radius fractures, respectively, peaked at 444 (25.52%) and 424 (26.72%) sites in 2017, which decreased to troughs of 351 (20.17%) and 298 (18.78%) sites in 2019 (Supplemental Fig. 1).

Concerning the remaining fractures, fractures of the shaft of ulna occurred in 1094 sites (7.82%), shaft of radius 1083 sites (7.75%), proximal ulna 355 sites (2.54%), shaft of tibia 335 sites (2.40%), shaft of femur 326 sites (2.33%), distal tibia 284 sites (2.03%), proximal radius 268 sites (1.92%), skull 195 sites (1.39%), proximal humerus 187 sites (1.34%), distal fibula 185 sites (1.32%), shaft of fibula 185 sites (1.32%), phalanges of toes 177 sites (1.27%), clavicle 156 sites (1.12%), pelvis 143 sites (1.02%), shaft of humerus 112 sites (0.80%), metatarsal bones 111 sites (0.79%), metacarpal bones 77 sites (0.55%), distal femur 76 Sites (0.54%), proximal femur 72 sites (0.51%), tarsal bones 68 sites (0.49%), ribs 66 sites (0.47%), vertebrae 59 sites (0.42%), proximal tibia 52 sites (0.37%), patella 15 sites (0.11%), scapula 9 sites (0.06%), proximal fibula 7 sites (0.05%), carpal bones 2 sites (0.01%), and sternum 1 site (0.007%) (Fig. 1).

Epiphyseal injuries are a unique type of pediatric fracture. Among the 13,982 fracture sites, epiphyseal injuries occurred in 1209 (8.65%) sites. The most common epiphyseal injury was to the distal radius (405 sites), accounting for 33.50% of all epiphyseal fractures. Moreover, fractures of the phalanges of fingers, distal humerus, distal tibia, and distal ulna occurred in 266 (22.00%), 163 (13.48%), 148 (12.24%), and 51 (4.22%) sites, respectively (Supplemental Table 1).

### Complications and multisystem injuries

In terms of complications, concomitant nerve damage occurred in 327 cases (3.12%), with the most common being radial nerve injury (162 cases), which was frequently associated with distal humerus fractures (85 sites). The second most common injury was ulnar nerve injury, which occurred in 46 cases (14.07%) and was also frequently associated with distal humerus fracture (36 sites). The third most common was median nerve injury, which occurred in 39 cases (0.37%), with a similar frequent association with the distal humerus fracture (34 sites).

In this study, 75 cases (0.72%) of fractures with concomitant osteofascial compartment syndrome. The most common fractures that caused compartment syndrome were the distal tibial and metatarsal (12 sites each) fractures, followed by the distal fibula (10 sites), shaft of tibia (8 sites), phalanges of toes (7 sites), and metacarpal (6 sites) fractures. The most common causes of injury leading to osteofascial compartment syndrome were car accidents (39 cases, 52.00%), followed by falls (10 cases, 13.33%) and crush injuries (9 cases, 12.00%) (Table 2).

**Table 2:** Fracture sites and injuries that cause compartment syndrome

Parameter	Patients N(%)
Fracture site	
Distal tibia	12 (13.79%)
Metatarsal bones	12 (13.79%)
Distal fibula	10 (11.49%)
Shaft of tibia	8 (9.20%)
Phalanges of toes	7 (8.05%)
Metacarpal bones	6 (6.90%)
Tarsal bones	5 (5.75%)
Distal humerus	5 (5.75%)
Phalanges of fingers	5 (5.75%)
Distal ulna	4 (4.60%)
Shaft of fibula	4 (4.60%)
Proximal tibia	3 (3.45%)
Shaft of radius	2 (2.30%)
Distal radius	2 (2.30%)
Proximal ulna	1 (1.15%)
Shaft of humerus	1 (1.15%)
Cause of injury	
Road traffic injuries-car accident	39 (52.00%)
Daily-life injuries-falls	10 (13.33%)
Daily-life injuries- crush injuries	9 (12.00%)
Road traffic injuries-bicycle-spoke injuries	3 (4.00%)
Daily-life injuries-falls from height	3 (4.00%)
Daily-life injuries-pinch injuries	3 (4.00%)
Daily-life injuries-twist injuries	2 (2.67%)
Daily-life injuries-sprains	2 (2.67%)
Sports injuries-basketball falls	2 (2.67%)
Road traffic injuries-bicycle falls	1 (1.33%)
Daily-life injuries-cuts	1 (1.33%)

Apart from musculoskeletal injuries, there were 258 cases with concomitant injuries to other systems (2.46%), with the most common cause of injury being road traffic accidents (188 cases, 72.87%), followed by falls from height (63 cases, 24.42%). Concomitant neurological injuries were the most common (138 cases, 53.49%), and the main concomitant fracture site was the skull (134 sites, 97.10%). Moreover, the numbers of cases of concomitant respiratory, digestive, and urinary system injuries were 122 (47.29%), 86 (33.33%), and 48 (18.60%), respectively.

Of 258 pediatric patients with multisystem injuries, 23 (8.91%) had concomitant shock. The most common cause of injury leading to shock was car accident (11 cases, 47.83%), followed by falls from height (10 cases, 43.48%), bunk bed fall (1 case, 4.35%), and machine twist injury (1 case, 4.35%). Of the 23 pediatric patients with shock, the most common concomitant fracture sites were the pelvic ring and ribs (12 sites each, 52.17%), followed by the vertebrae (11 sites, 47.83%), skull (9 sites, 39.13%), and scapula and shaft of tibia (3 sites each, 26.09%). Furthermore, there were 5 (21.74%) open fracture sites among these 23 pediatric patients with multisystem injuries and concomitant shock (Table 3).

**Table 3:** The fracture site that caused shock

Parameter	Patients N(%)
Pelvis	12 (15.38%)
Rib	12 (15.38%)
Vertebrae	11 (14.10%)
Skull	9 (11.54%)
Scapula	3 (3.85%)
Shaft of tibia	3 (3.85%)
Proximal ulna	2 (2.56%)
Distal ulna	2 (2.56%)
Shaft of fibula	2 (2.56%)
Shaft of humerus	2 (2.56%)
Proximal humerus	2 (2.56%)
Distal humerus	2 (2.56%)
Shaft of femur	2 (2.56%)
Distal femur	2 (2.56%)
Distal radius	2 (2.56%)
Shaft of radius	2 (1.28%)
Clavicle	2 (2.56%)
Shaft of ulna	1 (1.28%)
Proximal femur	1 (1.28%)
Distal tibia	1 (1.28%)
Proximal radius	1 (1.28%)
Metatarsal bones	1 (1.28%)
Phalanges of toes	1 (1.28%)

### Fracture treatment methods

Of the 10,486 pediatric patients, 1295 (12.35%) and 9191 (87.65%) underwent non-surgical and surgical treatments, respectively. In the non-surgical treatment group, 1063 (82.05%) and 232 (17.92%) patients underwent external plaster fixation and fracture site immobilization, respectively. In the surgical treatment group, 2095 (22.79%) and 7096 (77.21%) patients underwent open and closed reductions, respectively. In the open reduction subgroup of the surgical treatment group, Kirschner wire fixation, screw fixation, intramedullary nailing, steel plate fixation, Wires nail, Traction, external bracket fixation and splint fixation were performed in 1141, 229, 191, 15, 15, 13, 3 and 1 patients, respectively. In the closed reduction subgroup of the surgical treatment group, plaster fixation, Kirschner wire fixation, intramedullary nailing, Traction, screw fixation, external bracket fixation, and splint fixation, were performed in 260, 5509, 497, 407, 128 cases, 9, and 2 patients, respectively (Table 4).

**Table 4:** Treatment and anesthesia of patients

Parameter	Patients
Non-surgical treatment	
Fracture site immobilization	232
External plaster fixation	1063
Open reduction subgroup	
Kirschner wire fixation	1141
Screw fixation	229
Intramedullary nailing	191
Wires nail	15
Steel plate fixation	15
Traction	13
External bracket fixation	3
Splint fixation	1
Closed reduction subgroup	
Kirschner wire fixation	5509
Intramedullary nailing	497
Traction	407
External plaster fixation	260
Screw fixation	128
External bracket fixation	9
Splint fixation	2
Open reduction subgroup	
Local anesthesia	68
General anesthesia	2027
Closed reduction subgroup	
Local anesthesia	437
General anesthesia	6659

In this study, the surgical treatment group included 7096 and 2095 patients who underwent closed and open reductions, respectively; thus, the ratio of closed to open reduction was 3:1. The number of closed reduction cases peaked at 1719 (24.22%) in 2017, and decreased to a trough of 1409 cases (19.86%) in 2019. The number of open reduction cases peaked at 558 (26.63%) in 2018, and then followed downward trend (Table 4).

For patients who underwent surgical treatment, the averages of the operation time, length of hospital stay, and total hospitalization cost were 42.91 ± 36.09 min, 5.22 ± 7.63 d, and RMB (8142.68 ± 11,140.46), respectively (Table 1). Of the 9191 pediatric patients who underwent surgical treatment, general and local anesthesia were performed in 8686 (94.51%) and 505 (5.49%) patients, respectively (Table 4).

**Fig. 3:**
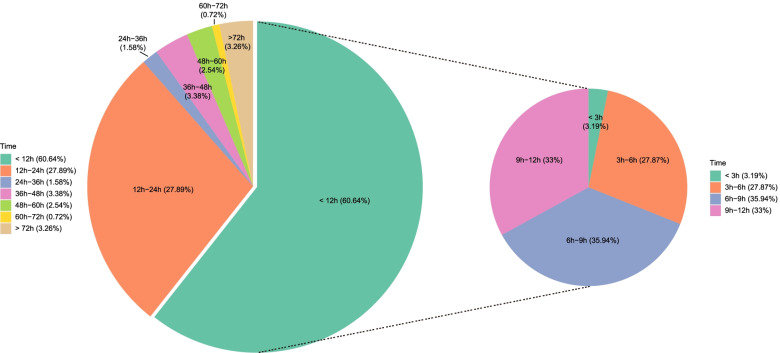
Interval time between injury and hospitalization. This picture expresses the interval time between the injury and the hospitalization in our hospital

Moreover, in the surgical treatment group, the intervals between hospital admission and surgical treatment were ≤ 12 h in 5584 (60.76%) patients, of which 178 were < 3 h; 3-6 h, 6-9 h, 9-12 h, 12-24 h, 24-36 h, 36-48 h, 48-60 h, 60-72 h, and > 72 h in 1553, 2003, 1839, 2563, 145, 311, 233, 66, and 300 patients, respectively (Fig. 3).

### Causes of fractures

Based on the first-level classification of injury causes, we observed daily-life injuries, road traffic, sports, birth, abuse, iatrogenic, and unknown causes of fracture in 8655 (82.54%), 989 (9.43%), 716 (6.83%), 75 (0.72%), 4 (0.04%), 3 (0.03%), and 44 (0.42%) patients, respectively.

According to the second-level classification of injury causes, falls represented the leading cause of fractures in children, with the number of cases reaching 7106 (82.10%). The number of falls in the infant group was 648 (9.12%), which peaked at 156 in 2018. The number of patients with falls in the preschool children group was 2997 (42.18%), which peaked at 749 in 2016, decreased, and increased again to 724 in 2018. The numbers of patients with falls in the school children and adolescent groups were, respectively, 2667 (37.53%) and 794 (11.17%), which respectively peaked at 632 in 2016 and 201 in 2019. Among all age groups, the number of patients with falls reached a peak of 1717 in 2018, and declined in subsequent years.

The second leading cause of injury was car accidents, affecting 650 (65.72%) patients, in whom the incidence was highest in the preschool children group (328 cases, 50.46%). The number of hospital visits for car accidents in the preschool children group peaked on Saturdays and Sundays, reaching 55 on both days; moreover, the peak visiting hour was 21:00, with 47 cases. The number of patients with car accidents reached a peak of 178 cases (27.38%) in 2017, and then gradually decreased in subsequent years. The third, fourth, and fifth leading causes of injury were clipping, crush injuries, and furniture-related falls, respectively, affecting 465 (5.37%), 343 (3.96%), and 327 (3.78%) patients (Supplemental Tables 2 and 3).

### Days and months of fracture occurrence

By retrospectively analyzing the 9544 pediatric patients admitted to our hospital over 4 calendar years from January 2016 to December 2019, we found that the peak time for fractures was on Saturdays and Sundays, with 3172 cases (33.24%). Moreover, the highest number of fractures occurred on Saturdays (1731 cases, 18.14%), followed by Sundays (1441 cases, 15.10%).

Furthermore, according to our study, the peak number of fractures occurred between July-November (4664 cases, 48.87%), with the highest number of fractures occurring in October (992 cases, 10.40%). The average temperature, rainfall, sunshine hours, and relative humidity for the month of October were (25.71 ± 0.70)°C, (131.23 ± 132.74) mm, (183.93 ± 29.61) h, and (71.51 ± 5.74)%, respectively (Supplemental Fig. 2).

Of the 9544 pediatric patients included during the 4-calendar-year period, the total number of patients in the infants and preschool children groups was 5081 (53.24%), the number of fractures peaked between July and October (2101 cases, 41.35%), and the highest number of fractures occurred in August (576 cases, 11.34%). In August, the average temperature, rainfall, sunshine hours, and relative humidity were (28.58 ± 0.55)°C, (436.54 ± 85.04) mm, (159.69 ± 40.44) h, and (83.63 ± 2.32)%, respectively. The total number of patients in the school children and adolescent groups was 4463 (46.76%), the number of fractures peaked between July and November (2164 cases, 48.49%), and the highest number of fractures occurred in October (459, 10.28%). In October, the average temperature, rainfall, sunshine hours, and relative humidity were (25.73 ± 0.68)°C, (126.67 ± 129.91) mm, s (185.01 ± 29.34) h, and (71.37 ± 5.60)%, respectively (Supplemental Fig. 2).

### Interval between injury and hospital visit and geographical location of injury

In our study population, the interval between injury and hospital admission was < 4 h, 4-6 h, 7-11 h, 12-23 h, 24-47 h, 48-71 h, 72 h-5 h, and 6-14 d in 4661 (44.45%), 3413 (32.55%), 590 (5.63%), 449 (4.28%), 444 (4.23%), 164 (1.56%), 275 (2.62%), and 490 (4.67%) patients, respectively.

A total of 765 patients (7.30%) had an interval between injury and hospital visit of > 72 h, and the main reasons for this delay were transfers to our hospital due to unsatisfactory treatment results in other hospitals (606, 79.22%), unsatisfactory results of outpatient conservative treatment (62, 8.10%), cases of high-energy trauma (car accident injuries and falls from height) that were transferred to our hospital after prior hemodynamic resuscitation (39, 5.10%), the lack of parental care for the patient after injury (23, 3.01%), parent refusal of hospitalization for surgical treatment at first visit (19, 2.48%), and neonatal patients who visited our hospital due to other chief complaints and were diagnosed with fractures during physical examination by a physician (16, 2.09%).

**Fig. 4:**
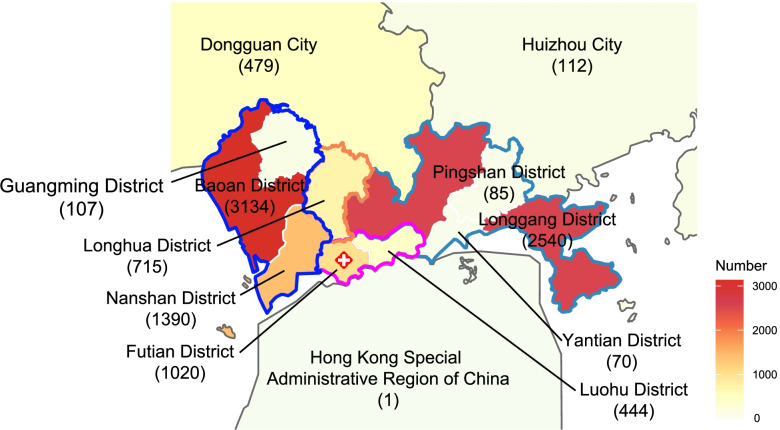
Geographic location of all inpatients. This picture expresses the geographical distribution of the number of fractures in Shenzhen

In our study, the highest number of fractures occurred in western Shenzhen with 4631 cases (44.16%), followed by eastern Shenzhen, southern Shenzhen, northern Shenzhen, and Shenzhen City, with 2713 (25.87%), 1464 (13.96%), 715 (6.82%), and 371 (3.54%) patients, respectively. Among the surrounding cities, Dongguan City, had the highest number of fractures with 479 cases (4.57%), followed by Huizhou City and the Hong Kong Special Administrative Region of China with 112 (1.07%) and 1 (0.001%) case, respectively (Fig. 4).

### Time of fracture

We retrospectively analyzed the time of hospital admission among the 9544 children treated at our hospital over the 4 calendar years from January 2016 to December 2019, and found that 4339 (45.46%) patients were admitted between 20:00 and 00:00. The number of hospital visits began increasing at 20:00, reaching a peak of 934 (21.53%) patients at 22:00, then began decreasing at 00:00 to reach a trough of 10 (0.10%) patients at 07:00 (Supplemental Fig. 3).

Among the 4339 patients admitted between 20:00 and 00:00, fracture injuries most frequently occurred in Baoan District (1373 cases, 31.64%), followed by Longgang District (1067 cases, 24.59%), and Nanshan District (565 cases, 13.02%) (Supplemental Fig. 3).

Among these 4339 pediatric patients, 605 (13.94%) had their initial visit at our hospital, and 3734 (86.06%) had their initial visit in another hospital. Among the 605 patients who initially visited our hospital, 154 (25.45%), 134 (22.15%), 120 (19.83%), and 94 (15.54%) patients were from Futian, Longgang, Baoan, and Nanshan Districts, respectively. Among the 3734 patients who were transferred to our hospital after their initial visit in local hospitals, 1253 (33.56%), 933 (24.99%), 471 (12.61%), and 227 (6.08%) patients had their initial visits at Shenzhen Baoan, Shenzhen Longgang, Shenzhen Nanshan, and Shenzhen Futian District Hospitals, respectively (Supplemental Fig. 3).

Among the 4339 patients admitted between 20:00 and 00:00, high-energy trauma (car accidents and falls from height) was the cause of injury in 287 (6.61%) patients. Twenty-nine (10.10%) patients with high-energy trauma had their initial visit at our hospital; of these, 7 (24.14%), 7 (24.14%), and 5 (17.24%) were from Longgang, Futian, and Luohu Districts, respectively. Local hospitals referred 258 (89.90%) patients to our hospital for specialist treatment; of these, 87 (33.72%), 70 (27.13%), and 20 (7.75%) had their initial visits at Shenzhen Baoan, Shenzhen Longgang, and Shenzhen Longhua District Hospitals, respectively.

## Discussion

The present study revealed many salient findings. A) First, more boys than girls were hospitalized for pediatric fractures, and the highest number of fractures occurred in children aged 3-6 years. B) Second, the most common fractures involved the distal humerus and resulted from falls; moreover, the most common epiphyseal injury involved the distal radius. C) Third, the most common concomitant nerve injury was radial nerve injury; furthermore, the most common concomitant multisystem injury was nervous system injury. D) Fourth, 11.40% of pediatric patients had two or more concomitant fracture sites, and 2.46% had multisystem injuries, including 23 patients with concomitant shock. E) Fifth, the treatment of fractures mainly involved surgical treatment by closed reduction. F) Sixth, of the 9191 patients in the surgical treatment group, 5584 received surgical treatment within 12 h of admission. G) Lastly, fractures occurred more frequently between July and November, and on Saturdays and Sundays. The peak hours of admission were from 20:00 to 00:00, and 7.30% of patients visited our hospital visit > 72 h post-injury. Between 20:00 and 00:00, 86.06% of patients were referred to our hospital after their initial visit at a local hospital.

### More educational programs on safety measures should be organized

A study by Rennie et al. [7] found that fractures in children aged 0-16 years occurred most frequently between the ages of 5-11 years, which accounted for 51.3% of fractures in all age groups. However, the Chinese scholars Chen et al. [12] found that the highest incidence of fractures occurred at approximately 3 years of age. This is consistent with our findings on the age distribution of pediatric fractures wherein fractures occurred most frequently between the ages of 3-6 years (41.77%), followed by 7-11 years (34.54%). The discrepancy in the peak age group for fractures between China and other countries may be related to differences in the use of sports protective equipment and health education. Our findings suggest the need for improvements in the abovementioned areas for children in fracture-prone age groups.

Landin [13] showed that during the period from birth to 16 years, the incidence of fractures was 42% for boys and 27% for girls. Our study also found a significantly higher proportion of boys than girls. Hedström et al. [3] and Cooper et al. [14] found that the peak age for fractures in girls was 11-12 years, whereas that for boys was 13-14 years; however, the peak for fractures in our study, for both boys and girls, was 3-6 years. Rennie et al. [7] showed that the incidence of fractures increased with age in both boys and girls before the age of 11 years; nonetheless, the incidence in girls began to decline after the age of 5 years, whereas that in boys continued to increase and was about twice that of girls by the age of 13 years. Our findings corroborated with those of Rennie et al., as we found that the number of fractures increased in boys but decreased in girls after 11 years, with a male to female ratio of 5.7: 1 at 11 years. Thus, despite their larger and stronger bones, boys have a higher incidence of fractures than girls, which may be attributed to their preference for more intense, competitive, and confrontational activities. Therefore, health education should place more emphasis on safety education for boys, especially those who are active and like to participate in high-risk sports that can lead to serious injuries; in addition, more attention should be placed on children aged 3-6 years.

Some studies have suggested that major pediatric fracture sites vary with age. More specifically, clavicular, distal humeral, distal radial, and metacarpal fractures are the most common in children aged < 1 year, 1-3 years, 4-14 years, and 15-16 years, respectively [9]. However, in the present study, distal humeral and distal radial fractures were most common in children aged < 11 years and 12-18 years, respectively.

Brudvik et al. [15] showed that pediatric fractures occur primarily in the upper limbs with the distal forearm as the most common fracture site. Children have a habit of extending their arms to protect themselves when they fall, which is the main reason why they are more prone to having upper limb fractures [11]. Among the 13,982 fracture sites in this study, there were 10,269 long bone fractures of the upper limb, accounting for 73.44% of all fracture sites. The distal humerus was the most common fracture site (3843 cases, 27.50%); however, the number of distal forearm fractures was 3327 cases, accounting for 23.79% of all fracture sites. Thus, our conclusions differ from the abovementioned reports in the literature on the common sites for upper limb fractures. In addition, elbow fractures account for 40-48% of upper limb fractures [16]. Behdad et al. [17] reported an epidemiological study of elbow fractures in Iranian children wherein supracondylar humeral fractures were the most common (58.0%). Similarly, our study included 4466 cases of elbow fractures, accounting for 43.50% of long bone fractures of the upper limbs.

Fractures of the phalanges of fingers are a common type of pediatric fractures resulting from trauma in emergency departments and outpatient clinics, with a high annual incidence [18–21]. A survey found a very low annual incidence of hand fractures among toddlers in the United Kingdom (34/100,000 children); nevertheless, this figure increases by approximately 20-fold after the age of 10 years, reaching 663/100,000 in children aged 11-18 years [21]. Many previous studies revealed that hand fractures were more common in boys than in girls, with 65-75% of fractures occurring in boys and a peak occurring around 9-14 years [22–24]. However, among the 1112 cases of fractured phalanges of fingers in this study, which included 696 boys and 416 girls, the peak and trough ages of fracture occurrence were 3-6 years (469 cases) and 12-19 years (120 cases), respectively. This finding differed from that previously reported in the literature in other countries.

Epiphyseal fractures are a unique type of pediatric fractures, accounting for 15-30% of all pediatric fractures. The incidence of epiphyseal fractures is higher in boys than in girls, and is more common in the upper limbs [25, 26]. Komura et al. [27] found that, of all epiphyseal fractures, those of the distal radius are relatively common. Steinberg et al. [28] showed that about 50% of epiphyseal fractures occurred in the proximal radius. Peterson et al. [29] found that the most common site of epiphyseal fracture was the phalanges of the fingers. In the present study, epiphyseal fractures occurred in 1209 cases, accounting for 8.65% of the total number of fractures; moreover, 73.20% of all epiphyseal fractures occurred in the upper limb. The most common type of epiphyseal fracture was that of the distal radius, which occurred in 405 cases and accounted for 33.50% of all epiphyseal fractures. This was followed by epiphyseal fractures of the phalanges of fingers. Therefore, in terms of the location and type of epiphyseal fractures, we should focus on the prevention of upper limb pediatric fractures, especially those of the distal humerus and phalanges of fingers. Fractures of the distal radius should be examined in detail for concomitant epiphyseal injuries. In children with suspected epiphyseal plate injuries, it is important to prevent serious complications such as bone discontinuity and growth disorders due to delayed diagnosis and treatment, misdiagnosis, or missed diagnosis.

Cheng et al. found an increase in the frequency of closed reduction and percutaneous pinning in pediatric fractures, a corresponding decrease in open reduction, and a significant reduction in the length of hospital stay [11]. In the present study, the number of closed reductions peaked in 2017 (1719 cases), and subsequently decreased to its trough in 2019 (1409 cases). The number of open reductions peaked in 2018 (558 cases), and then decreased subsequently. In this study, 12.35% of patients with pediatric fractures underwent conservative treatment.

Although there are multiple causes of pediatric fractures, falls during normal playing and sports represent a major cause. Both Gouiding et al. [30] and Mansoor et al. [31] identified play-related falls as a primary cause of injury. Similarly, our study identified falls as the most common cause of injury, accounting for 67.77% of all causes. It is also the most common cause of injury in all age groups, with the highest number of reported cases of injury in children aged 3-6 years. Children in this age group are able to move independently, and often trip and fall while playing.

A study conducted by Osmond et al. [32] concluded that road traffic injuries represented a common cause of severe trauma in children, and this also varied across age groups. In this study, road traffic injuries included 650 and 211 cases involving car accidents and bicycle falls, respectively. Car accidents peaked at 3-6 years (328 cases), whereas bicycle falls peaked at 7-11 years (85 cases). Such high-energy traumas often lead to open fractures, polytrauma, and shock, which pose a serious threat to children’s health. Therefore, parents, the community, and schools, should be educated on traffic safety, including the use of car safety seats, wearing protective gear while riding bicycles, and increased supervision [33, 34].

With respect to bicycle-related fractures, attention should also be given to bicycle-spoke injuries, which occur when the limb comes into contact with the spokes of the bicycle wheel, thereby leading to entanglement of the limb in the spokes and crushing of the limb against the bicycle frame. Injuries are usually sustained by children who are bicycle passengers, and involve mainly lower limb soft tissue damage, followed by lower limb fractures [35, 36]. In this study, 103 cases of bicycle-spoke injury were observed, predominantly in children aged 3-6 years, and the most common fracture site was the distal tibia. Although bicycle-spoke injuries are usually not life-threatening, the resulting socioeconomic damage is substantial. We believe that, in order to prevent bicycle-spoke injuries, safety education for guardians should be vigorously promoted, as this enhances supervision and prevention. With the growing popularity of bicycle sharing, bicycle manufacturers should improve the design and structural defects of the bicycle itself. This can be done by installing protective equipment (for instance, protective nets) to preventing toes from getting caught between spokes and designing special safety child backseats.

In this study, falls from height affected 114 patients, and peaked in children aged 3-6 years (50 cases). We believe that emphasis should be placed on the protection of high-rise buildings, and promoting safety education for guardians of children in the peak age group of 3-6 years. For example, in New York, a program themed “Children Can’t Fly” was organized to prevent fall injuries. Through extensive public safety health education, the installation of guardrails on high-rise windows, and the installation of protective nets, remarkable results were achieved in the number of fall injury victims, which dropped by 50% after 3 years, and by 96% after 7 years [37].

The third leading cause of injury in this study was clipping (465 cases). Door-clipping were observed in 403 patients, which primarily resulted in fractures of the phalanges of fingers. Al-Anazi [38] and Doraiswamy [39] concluded that door-related finger crush injury is the leading cause of finger injury in children. These injuries frequently occur at home, where the affected fingers are often crushed against the hinged side of the door, and are more likely to occur in younger children. The majority of door-clipping occur in the presence of an adult, thus highlighting the need for preventive measures. We should increase guardians’ awareness of these injuries and provide more educational programs on safety measures. Moreover, since fingers are most likely crushed in the hinged side of the door, finger guards can be installed to prevent finger entrapment. Furthermore, triangular rubber and plastic or wooden door wedges can be inserted into the bottom of the door to prevent its automatic closure.

There is a distinct seasonal pattern in the occurrence of pediatric fractures. Sinikumpu et al. [40] showed, in a study on fractures and weather, that most fractures occurred on dry days (79.7%) as opposed to rainy days, with a 3.5-fold higher risk of fracture on dry days. In a study conducted in Ireland, Masterson et al. [41] also showed a significant positive correlation between the number of sunshine hours per month and the corresponding number of hospital admissions for fractures, as well as a weak negative correlation between the number of fractures and the amount of rainfall per month; similar studies have been conducted in countries with summer holiday customs [42]. Fractures predominantly occurred in the afternoon, possibly due to the after-class or after-school hours during which schoolchildren are prone to fractures [43]. The high incidence of fractures in summer and autumn, and the lower incidence in winter, were also confirmed in the present study. This may be related to the favorable climate and temperature in summer and autumn, the long sunshine hours, the increased outdoor activity of children, and the use of thinner and fewer clothing that neither absorbs collision energy nor provides cushioning in the event of an accident. In addition, our study showed that July to November were the peak months for fractures. Apart from the aforementioned reasons, this may also be related to the National Day holiday and summer vacation in China, during which children spend more time outdoors and are exposed to accidental injuries and fractures. Therefore, on the one hand, medical resources need to be increased to ensure the priority and quality of care for children with fractures during this peak period. On the other hand, health education and promotion should be carried out in conjunction with schools and communities prior to this peak period.

### A closed-loop regional pediatric trauma treatment system should be established

This study documented that in 1373 (13.09%) patients, the interval between injury and visit to our hospital was greater than 24 h, of which 765 cases (7.30%) had an interval > 72 h. According to the detailed medical records, 606 (79.22%) patients were transferred to our hospital due to unsatisfactory results of inpatient treatment at their local hospitals, none of which were children’s specialized hospitals. Disparities exist in the treatment plans for fractures in children and adults, and our findings suggested that more children’s specialized hospitals or PTCs in general hospitals should be established in the region, in order to provide more timely and effective treatment plans for children with fractures.

### A comprehensive pediatric trauma treatment teams that follow a multidisciplinary treatment model should be substantively operated

In addition to injuries to the musculoskeletal system, there were 258 patients with concomitant injuries to other systems, most commonly involving neurological, respiratory, and digestive injuries. More so, it is challenging to treat high-energy injuries such as road traffic injuries and fall injuries. Twenty-three patients in our study had concomitant shock. An effective trauma care system ameliorate the prognosis of patients with trauma more than the individual clinical experiences of physicians. The findings of a meta-analysis showed that a well-developed emergency trauma system reduces overall trauma mortality by 15% [44]. An integrated model of care is best suited to children with multiple injuries, as it provides a stable and specialized trauma team responsible for every aspect of care [45]. The establishment and volition of the American trauma system over the past 40 years has achieved the standardization of trauma care through trauma centers, which has greatly helped reduce the impact of trauma on people of all ages [46]. We believe that we should learn from the successes of European and American countries in order to strengthen the construction of the pediatric trauma treatment system, the core of which should be the hierarchical construction of trauma centers, with an emphasis on the multidisciplinary integrated treatment of polytrauma and critical trauma patients. The biggest advantage of this approach is that the information platform, treatment process, staffing, and equipment supply are based on the principles of fastest efficiency and optimal process. This eliminates the previous practice of temporarily inviting relevant specialties for consultation in response to the patient’s condition and evolution, thus greatly reducing the rescue time and improving the treatment outcome. Furthermore, among the 9191 patients who underwent surgical treatment, 5584 (60.76%) were operated upon within 12 h of admission, which shows that the substantively operating comprehensive pediatric treatment team implemented by the our hospital has begun to bear fruit. This process specifically involves dispatching personnel from the orthopedic department to set up an emergency pediatric fracture specialty in the emergency department for the initial diagnosis and treatment of pediatric patients, setting up an inpatient department with beds, working with other departments of pediatric medicine and pediatric surgery to improve the relevant treatment plan, and coordinating with the surgical anesthesiology department for pediatric patients with indications for surgery in order to complete the relevant pre-operative preparations within 12 h.

## Conclusions

With regard to the pediatric population, we should strengthen safety education and accordance with their age, sex, cause of injury, fracture site, and other characteristics; increase protective measures for children’s activities. Moreover, a multidisciplinary treatment model based on PTCs should be established. In order to reduce the mortality and disability of children with trauma. This will positively influence the protection of lives and ameliorate the health of children.

## Limitation

We have conducted retrospective analysis based on the electronic medical record system and the outpatient medical record system, so we cannot know the AO classification, ISS score, Trauma score, open fracture classification, social deprivation and other data of children with fractures. In addition, the code of the injury cause and surgery method cannot be directly obtained from our original database, so it is still difficult to further standardize the cause of injury and surgical method into ICD-11 and ICD-9-CM-3. For the establishment of PTCs, although our data cannot provide strong support, we still hope to establish PTCs reference for some developing countries. But these are the limitation of our research.

## Supplementary Information

**Additional file 1: Supplemental Figure 1.** Number of pediatric fractures changes with the year. This picture illustrates the characteristics of fracture sites in various age groups in 2016-2019.

**Additional file 2: Supplemental Figure 2.** The exact month and week of pediatric fractures. This picture illustrates the characteristics of the data distribution of admission of patients in (a) different weeks and (b) different months.

**Additional file 3: Supplemental Figure 3.** The exact hour of pediatric fractures. This picture illustrates the data for occurrence time of fractures in patients.

**Additional file 4: Supplemental Table 1.** Epidemiological characteristics of epiphyseal fractures.

**Additional file 5: Supplemental Table 2.** The epidemiology of age group according to different etiologies.

**Additional file 6: Supplemental Table 3.** Changes in the causes of injuries in different years.

## Data Availability

The data sets used and analysed during the current study are available from these corresponding authors on reasonable request.

## Abbreviations

USA: United States of America
PTCs: pediatric trauma centers
PICU: pediatric intensive care unit

## References

[1] Jones G, Cooley HM (2002). Symptomatic fracture incidence in those under 50 years of age in southern Tasmania. J Paediatr Child Health.

[2] Mattila V, Parkkari J, Kannus P, Rimpelä A (2004). Occurrence and risk factors of unintentional injuries among 12- to 18-year-old Finns--a survey of 8219 adolescents. Eur J Epidemiol.

[3] Hedström EM, Svensson O, Bergström U, Michno P (2010). Epidemiology of fractures in children and adolescents. Acta Orthop.

[4] Lempesis V, Rosengren BE, Nilsson JÅ, Landin L, Tiderius CJ, Karlsson MK (2017). Time trends in pediatric fracture incidence in Sweden during the period 1950-2006. Acta Orthop.

[5] Joeris A, Lutz N, Wicki B, Slongo T, Audigé L (2014). An epidemiological evaluation of pediatric long bone fractures - a retrospective cohort study of 2716 patients from two Swiss tertiary pediatric hospitals. BMC Pediatr.

[6] Meling T, Harboe K, Søreide K (2009). Incidence of traumatic long-bone fractures requiring in-hospital management: a prospective age- and gender-specific analysis of 4890 fractures. Injury.

[7] Rennie L, Court-Brown CM, Mok JY, Beattie TF (2007). The epidemiology of fractures in children. Injury.

[8] Lyons RA, Sellstrom E, Delahunty AM, Loeb M, Varilo S (2000). Incidence and cause of fractures in European districts. Arch Dis Child.

[9] Jones IE, Williams SM, Dow N, Goulding A (2002). How many children remain fracture-free during growth? a longitudinal study of children and adolescents participating in the Dunedin Multidisciplinary Health and Development Study. Osteoporos Int.

[10] Hussain S, Dar T, Beigh AQ, Dhar S, Ahad H, Hussain I (2015). Pattern and epidemiology of pediatric musculoskeletal injuries in Kashmir valley, a retrospective single-center study of 1467 patients. J Pediatr Orthop B.

[11] Cheng JC, Shen WY (1993). Limb fracture pattern in different pediatric age groups: a study of 3,350 children. J Orthop Trauma.

[12] Cheng BC, Cai QX (1997). Statistical analysis of 3271 cases of fracture in children. Chin J Pediatr Surg.

[13] Landin LA (1997). Epidemiology of children's fractures. J Pediatr Orthop B.

[14] Cooper C, Dennison EM, Leufkens HG, Bishop N, van Staa TP (2004). Epidemiology of childhood fractures in Britain: a study using the general practice research database. J Bone Miner Res.

[15] Brudvik C, Hove LM (2003). Childhood fractures in Bergen, Norway: identifying high-risk groups and activities. J Pediatr Orthop.

[16] Rockwood CA, Wilkins KE, Beaty JH, Kasser JR, Skaggs DL, Flynn JM, et al. Rockwood and Wilkins' Fractures in Children, vol. 88, no. 10. Philadelphia: Wolters Kluwer; 2015. p. 2313–4.

[17] Landin LA, Danielsson LG (1986). Elbow fractures in children. An epidemiological analysis of 589 cases. Acta Orthop Scand.

[18] Naranje SM, Erali RA, Warner WC, Sawyer JR, Kelly DM (2016). Epidemiology of Pediatric Fractures Presenting to Emergency Departments in the United States. J Pediatr Orthop.

[19] Chung KC, Spilson SV (2001). The frequency and epidemiology of hand and forearm fractures in the United States. J Hand Surg [Am].

[20] Nellans KW, Chung KC (2013). Pediatric hand fractures. Hand Clin.

[21] Vadivelu R, Dias JJ, Burke FD, Stanton J (2006). Hand injuries in children: a prospective study. J Pediatr Orthop.

[22] Mahabir RC, Kazemi AR, Cannon WG, Courtemanche DJ (2001). Pediatric hand fractures: a review. Pediatr Emerg Care.

[23] Bhende MS, Dandrea LA, Davis HW (1993). Hand injuries in children presenting to a pediatric emergency department. Ann Emerg Med.

[24] Chew EM, Chong AK (2012). Hand fractures in children: epidemiology and misdiagnosis in a tertiary referral hospital. J Hand Surg [Am].

[25] Mann DC, Rajmaira S (1990). Distribution of physeal and nonphyseal fractures in 2,650 long-bone fractures in children aged 0-16 years. J Pediatr Orthop.

[26] Mizuta T, Benson WM, Foster BK, Paterson DC, Morris LL (1987). Statistical analysis of the incidence of physeal injuries. J Pediatr Orthop.

[27] Komura S, Yokoi T, Nonomura H, Tanahashi H, Satake T, Watanabe N (2012). Incidence and characteristics of carpal fractures occurring concurrently with distal radius fractures. J Hand Surg [Am].

[28] Steinberg EL, Golomb D, Salama R, Wientroub S (1988). Radial head and neck fractures in children. J Pediatr Orthop.

[29] Peterson CA, Peterson HA (1972). Analysis of the incidence of injuries to the epiphyseal growth plate. J Trauma.

[30] Goulding A (2007). Risk factors for fractures in normally active children and adolescents. Med Sport Sci.

[31] Mansoor K, Shahnawaz S, Ahmad A, Arif MM, Hamza M (2015). Epidemiology of childhood fractures in the city of Karachi. J Ayub Med Coll Abbottabad.

[32] Osmond MH, Brennan-Barnes M, Shephard AL (2002). A 4-year review of severe pediatric trauma in eastern Ontario: a descriptive analysis. J Trauma.

[33] Winston FK, Durbin DR, Kallan MJ, Moll EK (2000). The danger of premature graduation to seat belts for young children. Pediatrics.

[34] Kahl H, Dortschy R, Ellsässer G (2007). Injuries among children and adolescents (1-17 years) and implementation of safety measures. Results of the nationwide German Health Interview and Examination Survey for Children and Adolescents (KiGGS). Bundesgesundheitsbl Gesundheitsforsch Gesundheitsschutz.

[35] Kramer WL, Haaring GJ (2011). Bicycle spoke-related injuries in children: emphasise prevention. Ned Tijdschr Geneeskd.

[36] Powell EC, Tanz RR (2000). Tykes and bikes: injuries associated with bicycle-towed child trailers and bicycle-mounted child seats. Arch Pediatr Adolesc Med.

[37] Barlow B, Niemirska M, Gandhi RP, Leblanc W (1983). Ten years of experience with falls from a height in children. J Pediatr Surg.

[38] Al-Anazi AF (2013). Fingertip injuries in paediatric patients - experiences at an emergency centre in Saudi Arabia. J Pak Med Assoc.

[39] Doraiswamy NV (1999). Childhood finger injuries and safeguards. Inj Prev.

[40] Sinikumpu JJ, Pokka T, Hyvönen H, Ruuhela R, Serlo W (2017). Supracondylar humerus fractures in children: the effect of weather conditions on their risk. Eur J Orthop Surg Traumatol.

[41] Masterson E, Borton D, O'Brien T (1993). Victims of our climate. Injury.

[42] Issin A, Kockara N, Oner A, Sahin V (2015). Epidemiologic Properties of Pediatric Fractures in a Metropolitan Area of Turkey. Medicine (Baltimore).

[43] Tiderius CJ, Landin L, Düppe H (1999). Decreasing incidence of fractures in children: an epidemiological analysis of 1,673 fractures in Malmö, Sweden, 1993-1994. Acta Orthop Scand.

[44] Celso B, Tepas J, Langland-Orban B, Pracht E, Papa L, Lottenberg L (2006). A systematic review and meta-analysis comparing outcome of severely injured patients treated in trauma centers following the establishment of trauma systems. J Trauma.

[45] Wang SY, Li YH, Chi GB, Xiao SY, Ozanne-Smith J, Stevenson M (2008). Injury-related fatalities in China: an under-recognised public-health problem. Lancet.

[46] Mullins RJ (1999). A historical perspective of trauma system development in the United States. J Trauma.

