# Supplementary figures and images for: Epidemiology and management of 10,486 pediatric fractures in Shenzhen: experience and lessons to be learnt
Source: BMC Pediatr. 2022 Mar 29;22:161. doi: 10.1186/s12887-022-03199-0 (PMC8962138; doi:10.1186/s12887-022-03199-0)

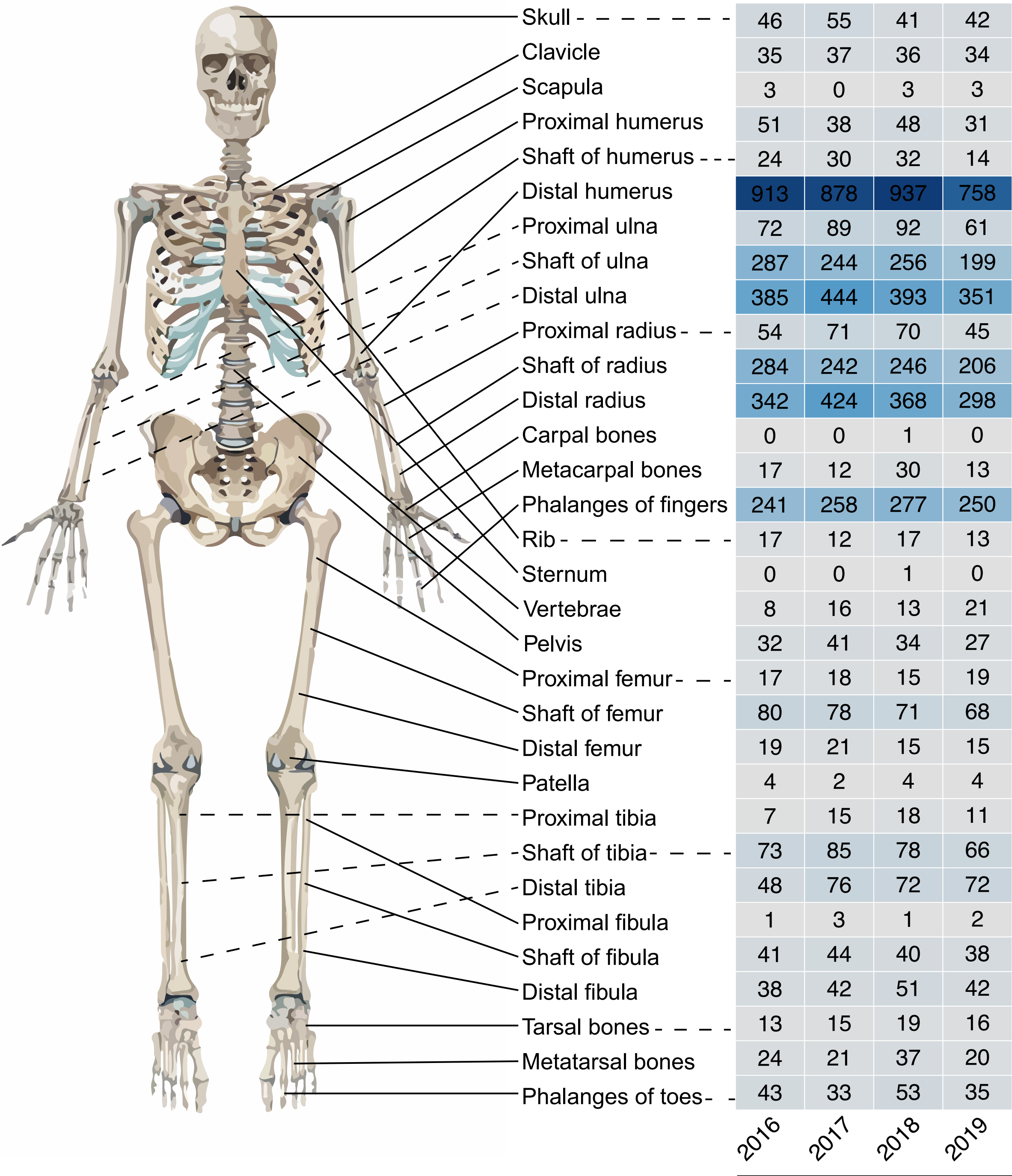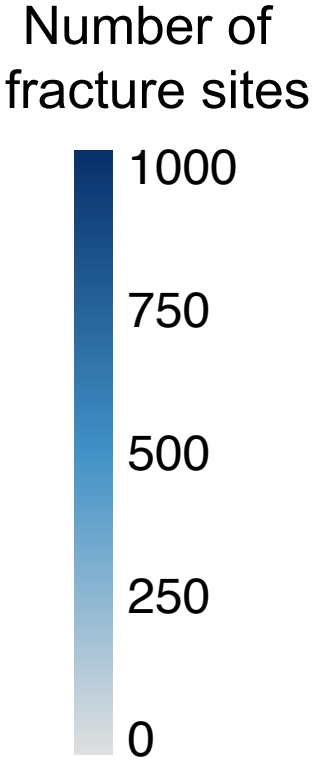

Years

Supplement: Supplementary file 1 — Additional file 1: Supplemental Figure 1. Number of pediatric fractures changes with the year. This picture illustrates the characteristics of fracture sites in various age groups in 2016-2019. [file 12887_2022_3199_MOESM1_ESM.pdf]

A

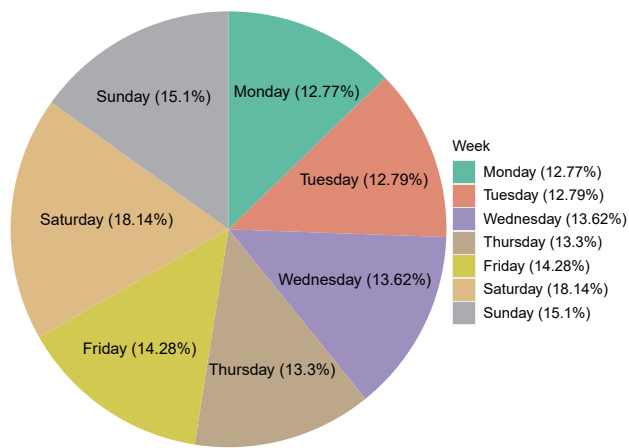

B

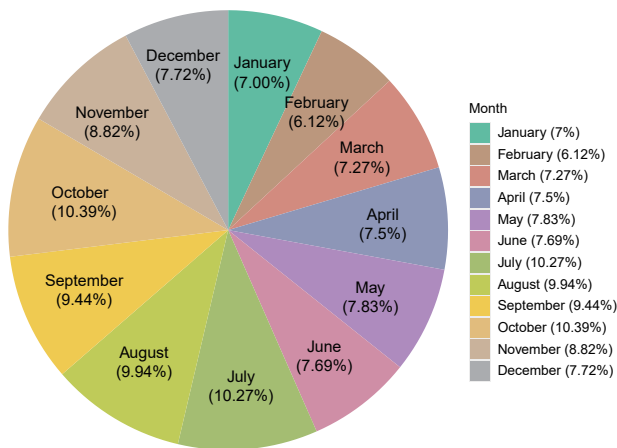

Supplement: Supplementary file 2 — Additional file 2: Supplemental Figure 2. The exact month and week of pediatric fractures. This picture illustrates the characteristics of the data distribution of admission of patients in (a) different weeks and (b) different months. [file 12887_2022_3199_MOESM2_ESM.pdf]

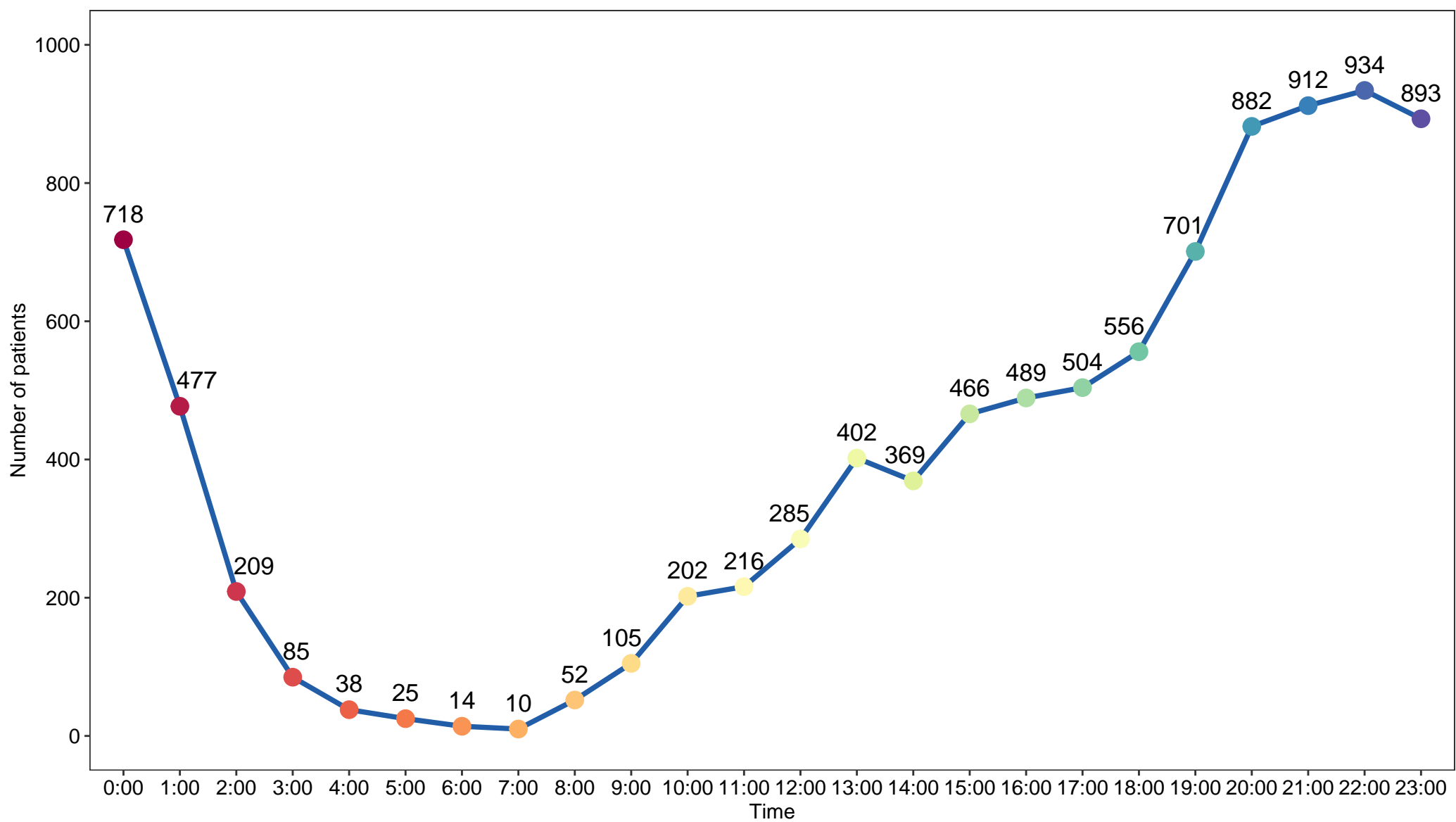

Supplement: Supplementary file 3 — Additional file 3: Supplemental Figure 3. The exact hour of pediatric fractures. This picture illustrates the data for occurrence time of fractures in patients. [file 12887_2022_3199_MOESM3_ESM.pdf]
