# Supplementary material for: Epidemiology and management of 10,486 pediatric fractures in Shenzhen: experience and lessons to be learnt
Source: BMC Pediatr. 2022 Mar 29;22:161. doi: 10.1186/s12887-022-03199-0 (PMC8962138; doi:10.1186/s12887-022-03199-0)
Supplement: Supplementary file 4 — Additional file 4: Supplemental Table 1. Epidemiological characteristics of epiphyseal fractures. [file 12887_2022_3199_MOESM4_ESM.pdf]

Supplemental table1. Epidemiological characteristics of epiphyseal fractures

Parameter	Patients n(%)
Distal radius	405(33.50%)
Phalanges of fingers	266(22.00%)
Distal humerus	163(13.48%)
Distal tibia	148(12.24%)
Distal ulna	51(4.22%)
Phalanges of toes	38(3.14%)
Distal fibula	28(2.32%)
Proximal humerus	24(1.99%)
Distal femur	22(1.82%)
Metatarsal bones	21(1.74%)
Proximal tibia	14(1.16%)
Metacarpal bones	13(1.08%)
Proximal femur	5(0.41%)
Proximal radius	4(0.33%)
Shaft of fibula	3(0.25%)
Proximal ulna	2(0.17%)
Shaft of tibia	2(0.17%)
