# Supplementary material for: Epidemiology and management of 10,486 pediatric fractures in Shenzhen: experience and lessons to be learnt
Source: BMC Pediatr. 2022 Mar 29;22:161. doi: 10.1186/s12887-022-03199-0 (PMC8962138; doi:10.1186/s12887-022-03199-0)
Supplement: Supplementary file 5 — Additional file 5: Supplemental Table 2. The epidemiology of age group according to different etiologies. [file 12887_2022_3199_MOESM5_ESM.pdf]

Supplemental table2. The epidemiology of age group according to different etiologies

Causes of fractures	Infants	Preschool child	School child	Adolescent
Road traffic injuries	80	481	303	125
Bicycle-spoke injuries	12	77	12	2
Falls from vehicles	0	16	7	2
Car accident injuries	66	328	199	57
Bicycle falls	2	60	85	64
Daily-life injuries	1042	3731	3001	881
Falls from height	8	50	38	18
Crush injuries	87	156	84	16
Furniture-related falls	103	171	45	8
Pinch injuries	139	236	78	12
Twist injuries	9	13	12	2
Sprains	2	10	10	16
Others	1	2	1	1
Strains	3	0	4	1
Cuts	39	86	58	12
Falls	648	2997	2667	794
Bunk bed falls	2	10	4	1
Bites	1	0	0	0
Sports injuries	10	152	305	249
Trampoline falls	0	5	4	2
Single bar falls	0	11	46	41
Slide falls	4	44	23	1
Skateboard falls	0	17	36	20
Fitness equipment falls	1	8	8	0
Basketball falls	1	1	21	82
Ice skating falls	0	15	46	8
Rocking horse falls	2	4	0	0
Falls while running	1	18	32	45
Balance bike falls	1	2	11	5
Swing falls	0	7	6	0
Ski falls	0	0	1	0

Parallel bar falls	0	1	0	0
Others	0	0	0	1
Taekwondo falls	0	1	10	5
Kick injuries	0	1	9	6
Dance falls	0	5	8	5
Playground falls	0	9	4	1
Falls during physical Education activities	0	0	12	14
Soccer falls	0	4	22	11
Jump rope falls	0	0	1	1
Swimming-related injuries	0	0	0	1
Martial arts falls	0	0	5	0
Birth injuries	75	0	0	0
Abuse injuries	0	1	3	0
Iatrogenic injuries	1	1	1	0
Unknown causes of fracture	19	14	9	2
