# Supplementary material for: Epidemiology and management of 10,486 pediatric fractures in Shenzhen: experience and lessons to be learnt
Source: BMC Pediatr. 2022 Mar 29;22:161. doi: 10.1186/s12887-022-03199-0 (PMC8962138; doi:10.1186/s12887-022-03199-0)
Supplement: Supplementary file 6 — Additional file 6: Supplemental Table 3. Changes in the causes of injuries in different years. [file 12887_2022_3199_MOESM6_ESM.pdf]

Supplemental table3. Changes in the causes of injuries in different years

Causes of fractures	2016	2017	2018	2019
Road traffic injuries	245	308	215	143
Bicycle-spoke injuries	22	32	31	13
Falls from vehicles	7	8	5	3
Car accident injuries	165	178	150	100
Bicycle falls	51	90	29	27
Daily-life injuries	2054	1873	2106	1808
Falls from height	34	24	20	27
Crush injuries	80	64	93	67
Furniture-related falls	63	107	79	59
Pinch injuries	115	116	109	96
Twist injuries	6	11	13	4
Sprains	6	5	10	14
Others	1	1	1	1
Strains	4	1	1	2
Cuts	38	30	60	54
Falls	1705	1504	1717	1482
Bunk bed falls	2	9	3	2
Bites	0	1	0	0
Sports injuries	101	253	199	123
Trampoline falls	2	3	4	2
Single bar falls	8	28	37	18
Slide falls	10	32	15	11
Skateboard falls	8	30	23	9
Fitness equipment falls	6	4	2	5
Basketball falls	10	28	33	27
Ice skating falls	12	35	12	7
Rocking horse falls	1	2	0	1
Falls while running	13	37	19	20
Balance bike falls	4	5	7	3
Swing falls	1	4	4	3
Ski falls	0	1	0	0

Parallel bar falls	0	1	0	0
Others	0	0	1	0
Taekwondo falls	3	4	4	3
Kick injuries	5	2	7	1
Dance falls	7	5	3	2
Playground falls	3	6	3	2
Falls during physical Education activities	5	11	8	2
Soccer falls	2	13	12	7
Jump rope falls	1	0	1	0
Swimming-related injuries	0	0	1	0
Martial arts falls	0	2	3	0
Birth injuries	20	22	15	10
Abuse injuries	3	1	0	0
Iatrogenic injuries	0	2	1	0
Unknown	12	11	10	9
